# Willingness to pay for one-stop anesthesia in pediatric day surgery

**DOI:** 10.1186/1824-7288-37-23

**Published:** 2011-05-17

**Authors:** Giovanni Mangia, Franco Bianco, Roberta Bonomo, Elisabetta Di Caro, Eufrasia Frattarelli, Paola Presutti

**Affiliations:** 1 Departement of Anestesiology, San Camillo Hospital, Rome, Italy

## Abstract

**Background:**

This study assesses the parents' Willingness To Pay (WTP) for One Stop Anesthesia (OSA). OSA is part of a free screening procedure that determines the timing of the anesthesiological assessment. In OSA-positive patients, the preoperative assessment is carried out on the same day as the surgery. The OSA allows patients who have to undergo surgery in a pediatric day surgery to avoid accessing the pre-admission clinic.

**Method:**

This is a descriptive cohort study. A sample of 106 parents were interviewed directly by means of a questionnaire. The questionnaire builds a hypothetical scenario where the interviewee has a chance to buy the OSA health service with the WTP. The WTP values are distributed in classes and are contingent to the market built in the questionnaire. The Chi Square and Cramer's V tests evaluate the WTP dependence on the parents' place of origin and occupation.

**Results:**

The approximate average of the WTP classes is €87.21 per family. The Chi Square test relative to the WTP classes and the places of origin is statistically significant (p < 0.05). The Cramer's V test is 0.347 and points to a positive association between the two demographics. The Cramer's V test of the WTP classes and the types of job is 0.339 and indicates a positive association.

**Conclusion:**

Nearly 90% of pediatric patients who were screened for timing the preoperative assessment are true positives to OSA. This allows doing away with the pre-hospitalization, with definite advantages for the families. This screening is a health service that families would be hypothetically willing to pay.

## Introduction

One-Stop Anesthesia (OSA) means carrying out the preoperative anesthesiological assessment of selected patients on the same day as their surgery [1]. The OSA is a free diagnostic screening of patients who have to undergo surgery where the anesthesiologist, sitting at his desk, decides the timing of the visit based on an anamnestic questionnaire. The questionnaire is drawn up by the surgeon who has required the intervention and who is going to operate the child. The visit takes place in a one-stop mode if the data are negative owing to personal and family pathologies or in a pre-hospitalization setting if a more detailed diagnosis is required (Care Pathway, Additional file 1). The OSA effectiveness has been described in a previous paper on diagnostic accuracy. The selection (a deskwork activity) is made with clinical data provided by the surgeon and has been compared to the anesthesiological assessment (gold standard). In this study, the true positives were 87.4% and the true negative 9%; therefore, the diagnostic accuracy was 96.4%. The potential limitation of this screening procedure, represented by the false positives (rare, being 1.4%), was only theoretical as all the patients have been operated on the same day after being subjected to examinations (e.g. cardiology consultation) and/or instrumental tests (e.g. ECG).

In Italy, only a few health-care facilities practice the one-stop methodology. More often than not, the anesthesiologist visits all patients, regardless of their medical history, 2-3 days before surgery (access to the pre-admission clinic in the day surgery unit). The willingness to pay (WTP) is the maximum price that a buyer can and wants to pay for a certain product or service, and is determined by the benefits that a consumer expects to receive in accordance with the consumer theory in economics [2,3].

This study purposes to measure the parents' WTP for the OSA with a view to ascertaining the monetary value of the free preoperative screening service used by the anesthesiologist to decide the timing of the preoperative assessment. This cohort study has made recourse to a questionnaire-based survey and direct interviews of parents and it has been used according to the contingent valuation methodology (CVM) [4,5].

## Materials and methods

The study was submitted to the Ethics Committee and to the informed consent of the parents. It was conducted for 45 consecutive days, during which 145 OSA-selected children underwent surgery. The WTP was obtained by conducting a questionnaire-assisted survey and direct interviews with parents on the occasion of the postoperative surgical control. The questionnaire-assisted survey was conducted using the contingent evaluation method that is frequently used to estimate the economic value of a product or a public service that is not traded in markets [6,7]. This method is based on the theory that, in order to estimate non-market values, individuals may be asked directly what monetary value they would be willing to pay for a product or an improvement project. The resulting WTP values are "contingent", or rather they depend on either the simulated market or the market constructed in the questionnaire [8].

### WTP questionnaire (Additional file 2)

An earlier pilot study on the WTP elicitation method has highlighted that parents prefer a close-ended question model with a checklist. The question about the WTP was preceded by a question about the *presumed savings*, which provided an answer in superimposable classes. The WTP checklist provided for five classes identified based on the expenses to be borne (transport costs, possible overnight stay), the loss of earnings (days of work lost) and intangible costs. This study has also revealed a low propensity of the parents to report their income and educational qualifications, so that such data were excluded from the questionnaire. Instead, employment data were obtained with no difficulty at all.

### Statistical analysis of the WTP

The interviewed sample consisted of 106 households and allowed calculating a 95% level of significance and a 5% sampling error. The interviewed parents were sampled by place of origin with respect to our day surgery facility that is located in the center of Rome. The statistical study was performed by means of a bi-varied analysis using the central values of the WTP classes [9]. The WTP was assessed in relation to the employment status of the parents and their place of origin. The employment status was divided into two groups. One group included parents who were both self-employed (professionals, artisans, entrepreneurs) or one parent who was self-employed and the other who was unemployed. The other group included parents who were both in (public or private) dependent employment or one parent who was in dependent employment and the other one who was unemployed. The place of origin of the patients was divided into two geographical areas: an area including Rome and its Province, and another one including the other cities and Provinces of the Lazio Region. The bi-varied analysis of the WTP was used for a comparison with both the qualitative characteristics (parents' occupation, place of origin) and the quantitative characteristics (estimated savings) in order to study the statistical dependence or independence.

The interdependence between WTP and place of origin was measured using the Chi Square and Cramer's V tests. The interdependence between WTP and parents' occupation was measured by means of the Cramer's V test. The association of WTP with the presumed savings was analyzed by calculating the linear correlation coefficient (Bravais-Person). The statistical analysis of data was carried out with open source software (PSSP, Free Software Foundation - GNU Project and OpenEpi, free software copyright ^© ^2003, 2008 Andrew G. Dean and Kevin M. Sullivan, Atlanta, GA, USA). The graphs were drawn up using the Statistica 4038 software available at: http://studiostat.unibocconi.it/fonti/software.html.

#### Additional aspects

The recourse to the questionnaire has allowed investigating a number of additional aspects. The distance measured in km between the patients' home and the hospital was calculated based on register data using a specific online software http://www.viamichelin.it. In those cases when both parents were employed, they were asked to specify the number of leaves with pay they had taken. Parents were also asked to give their overall opinion on the anesthesiological assessment with a number of answers ranging from poor, fair and good to excellent.

## Results

Table 1 shows the demographic characteristics of the sample. All the 106 parents with whom we got in contact accepted the interview. The approximate average of the WTP classes, end-point of the study, is €87.21. The approximate standard deviation of the classes is €64.97.

**Table 1:** Demographics of the sample

Demographics	Procedure	Cases	%
Age group	Less than 3 years	37	35
	From 3 to 6 years	17	16
	From 6 to 12 years	32	30
	From 12 to 26 years	20	19

Sex	Male	92	87
	Female	14	13

Place of origin	Rome and Rome Province	76	72
	Other Cities and Provinces in Lazio	30	28

Parents' job	Self-employed Couples (LA)*	18	17
	Employed Couples (LD)**	64	60
	Mixed Couples***	24	23

* Both parents are self-employed or one is self-employed and the other is unemployed

** Both parents are in dependent employment or one is employed the other is unemployed

*** One parent is self-employed and the other is in dependent employment

**Figure 1:**
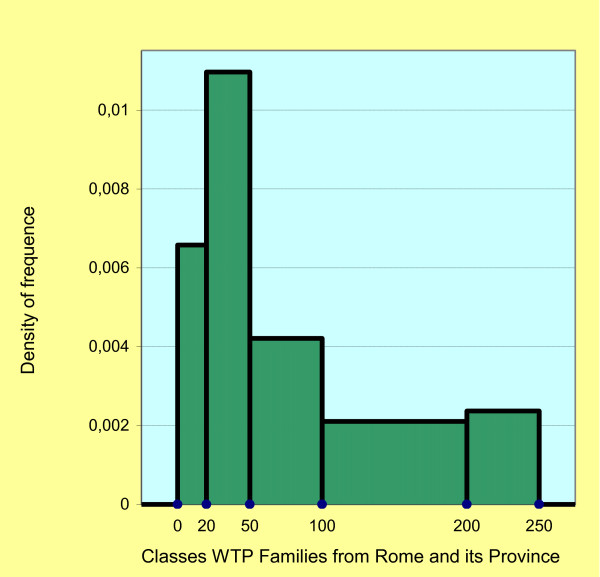
Histogram: density of frequence classes WTP families from Rome and its Province

**Figure 2:**
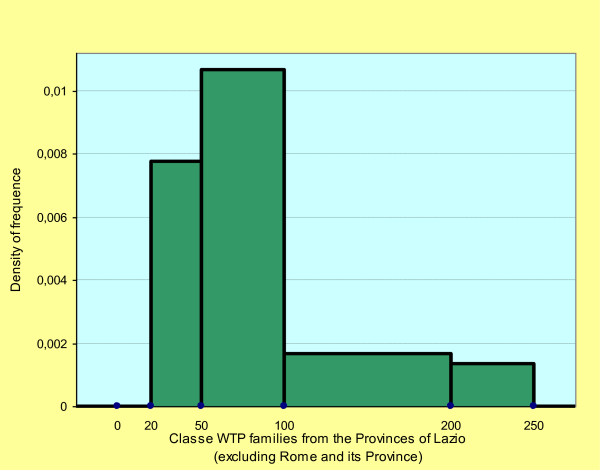
**Histogram: density of frequence classes WTP families from the Province of Lazio (excluding Rome and its Province)**.

The WTP varies if connected with other demographics. The connection of the WTP classes with the place of origin shows an approximate average of €83.88 for the households living in Rome and the Rome Province (Figure 1), and €86.5 for the households coming from the rest of the Lazio Region (Figure 2). The Chi Square test among the WTP classes and the places of origin is statistically significant (p < 0.05). The approximate standard deviation is €63.97 and €47.93, respectively. The Cramer's V test is equal to 0.347 and indicates a positive association between the two demographics.

**Figure 3:**
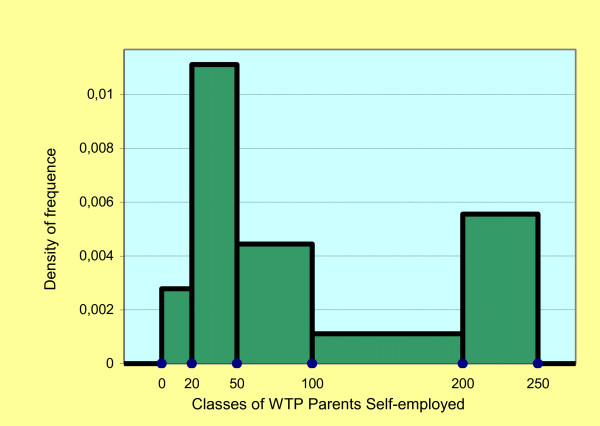
**Histogram: density of frequence classes WTP parents self-employed**.

**Figure 4:**
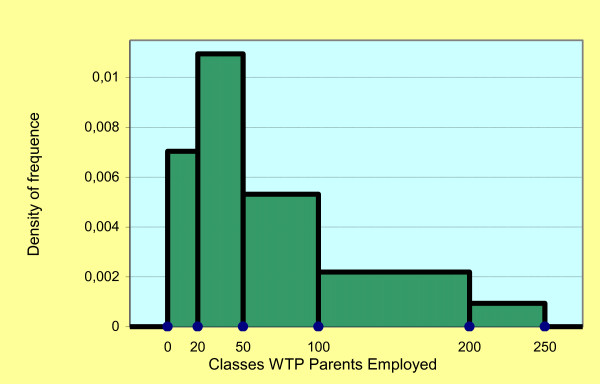
**Histogram: density of frequence classes WTP parents employed**.

The approximate average of the WTP classes based on the parent's occupation is €108.05 for the self-employed parents (Figure 3), €76.17 for the parents in dependent employment (Figure 4) and €101.04 for mixed couples. The approximate standard deviation is €83.28, €58.8 and €61.62, respectively. The Cramer's V test for the WTP between couples of self-employed parents and parents in dependent employment is equal to 0.339 and indicates a positive association between the two demographics.

The approximate average "presumed savings" are €97.69 and the linear correlation coefficient with the WTP is 0.861, pointing to a good dependence of the two characteristics.

### Additional data

Having saved themselves the trouble of going from their home to the hospital and back, the patients of the sample saved on average of 32 km if coming from the city of Rome, 95 km if coming from the Rome province, 134 km if coming from Latina or its province, 246 km if coming from Frosinone or its province, 172 km if coming from other provinces.

The days off from work saved by parents in dependent employment thanks to the OSA were approximately 98. The opinion of the parents on the one-stop selection ranged from good (31%) to excellent (69%).

## Discussion

The 106 parents interviewed are all aware of the savings that result from doing away with the need to return to the hospital after the OSA screening. The average monetary WTP value they attach to such a service is approximately €10 less than the "presumed costs" incurred. The two parameters are positively correlated. The highest WTP values are referred to by parents who are both self-employed and by those coming from outside Rome and its province. Such a connection is supported by a positive statistical dependence of the WTP. These results are important as parents pass a favorable judgment on the OSA particularly if they are self-employed and/or living far away from our day surgery unit. The WTP is strongly linked to income. The low propensity of parents to report their income and the lack of comparison with the WTP represent an important limitation of the study. Another limitation is represented by the False Positives to the OSA. So far, this aspect has only been hypothetical and, at any rate, concerns a negligible minority of the selected patients. No renunciation or cancellation of the operation, equal to 9 cases while the study was being carried out, occurred for the OSA selection. This aspect has been evaluated by phone contacts with parents who have either given up or cancelled the surgical operation. Anyway, in order to avoid a useless access and the cancellation of scheduled operations, the nurses contact the parents by phone 2-3 days before the procedure to get information about health conditions.

Sample surveys play a very significant role among the methods for determining the willingness to pay. In fact, the surveys allow detecting opinions on public goods, expressed directly by the interviewees. The CVM overcomes the problem of the lack of a market for the good under consideration by presenting a hypothetical market where the interviewee has a chance to "buy" the good.

The WTP is also used to estimate indirect and intangible costs [10]. Several indirect costs are borne when having access to a hospital. Indirect costs include transport costs and production losses that are borne by households, society and entrepreneurs. The intangible costs include the loss of free time, the stress caused by the trip, the waiting time and the examination. In other studies, the WTP was used to elicit the preferences of the patients and/or their families to avoid a few side effects of the anesthesia [11,12].

The OSA can be included among the one-stop shop procedures, provided in a single appointment to improve both efficiency and user satisfaction [13]. In the clinical field (one-stop clinics), these procedures are used for diagnostic screening, surgery for adult patients and pediatric patients [14-20]. In the field of anesthesia abroad, the recourse to preoperative screenings is quite common, since they reduce the access to the day surgery unit and allow a one-stop procedure [21]. In the UK, the nurses often deal with the assessment having recourse to questionnaires [22,23]. The surgeon invites the parents to fill in a detailed questionnaire about the medical history of their child. An experienced nurse examines the questionnaire and classifies the patients as suitable or unsuitable for day surgery and borderline cases. Anesthesiologists assess beforehand only borderline patients. In North America, telephone screenings are frequent [24]. This proves particularly useful if the surgeon had visited the child in a place far from the day surgery location. Just as the questionnaire-based method, the telephone screening can be used to invite borderline patients for a further assessment in the day surgery unit. In any event, the anesthesiologist visits all the patients on the same morning of the surgical operation.

The OSA methodology adopted in Italy, and the screenings adopted in the UK and in North America have a common objective: the reduction of the examinations of eligible patients and the assessment in the pre-admission clinic of just borderline patients. This allows reducing the number of times the families have to go to the day surgery unit, which is indeed the main purpose of the OSA. In Italy, the timing of the anesthesiological examination is a varied and controversial aspect [25,26]. Most day surgery facilities do not use a screening system for timing purposes. The patients are often visited in a pre-hospitalization setting and, in a few cases, they are hospitalized on the day before the operation or during the surgeon's first visit. These methods can generate unnecessary hospital accesses, inappropriate admissions and a waste of resources.

## Conclusion

The screenings used to determine the timing of the anesthesiological assessment cut down health-care costs and households appreciate these services, given that parents attach to them a monetary value and would be willing to pay for them.

## Conflicts of interests

The authors declare that they have no competing interests.

## Authors' contributions

All authors were involved in the conception of the study. GM carried out data extraction. He is the corresponding author of the paper. GM conducted statistical analysis and was checked for correctness by PP. GM drafted the paper with contributions from the co-authors. All authors read and approved the final manuscript prior to submission.

## Supplementary Material

Additional file 1 Care pathway timing anesthesia evaluation pre-operative Click here for file

Additional file 2 One-stop anesthesia evaluation questionnaire Click here for file
