# Supplementary figures and images for: Willingness to pay for one-stop anesthesia in pediatric day surgery
Source: Ital J Pediatr. 2011 May 17;37:23. doi: 10.1186/1824-7288-37-23 (PMC3121670; doi:10.1186/1824-7288-37-23)

**
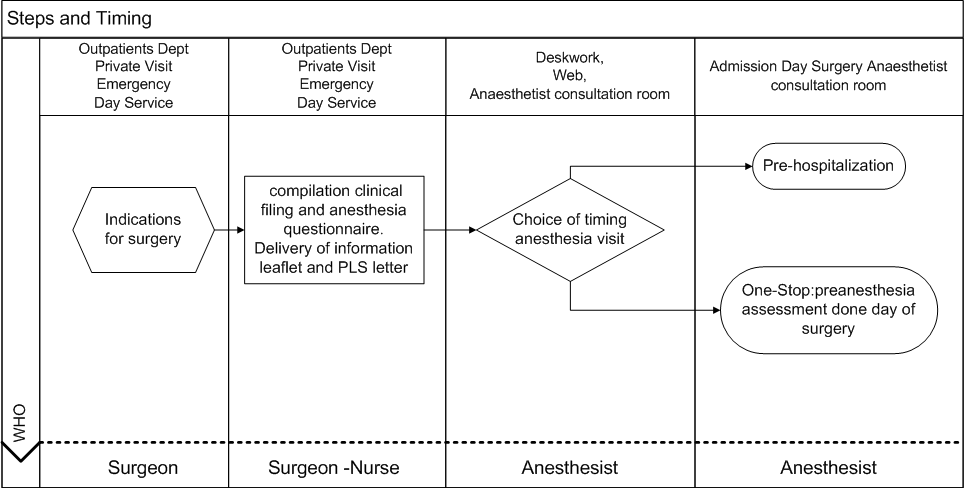
**

Supplement: Additional file 1 — Care pathway timing anesthesia evaluation pre-operative [file 1824-7288-37-23-S1.DOC]
