# Supplementary material for: Willingness to pay for one-stop anesthesia in pediatric day surgery
Source: Ital J Pediatr. 2011 May 17;37:23. doi: 10.1186/1824-7288-37-23 (PMC3121670; doi:10.1186/1824-7288-37-23)
Supplement: Additional file 2 — One-stop anesthesia evaluation questionnaire [file 1824-7288-37-23-S2.DOC]

**ONE-STOP ANESTHESIA EVALUATION QUESTIONNAIRE**

(To be administered to parents during the postoperative control visit)

- Demographic data. Personal data of the child (age, sex), family residence (Rome, Rome Province, Latina and its Province, Frosinone and its Province, other Provinces) and parents’ occupation (self-employed, dependent employment, unemployed).
- Parents’ opinion on the timing of the one-stop anesthesiological assessment.

Introductory remarks. After the evaluation of the information about the child’s physical conditions, collected by the surgeon during the preoperative evaluation, the anesthesiological assessment is performed without any problems on the same day as surgery. This method allows coming to the Hospital just once, since the anesthesiological assessment is performed on that day (on the morning of the procedure, by the anesthetist who administers the anesthesia) instead of twice (a few days before in the pre-admission clinic. and on the morning of the procedure probably by a different anesthetist). This method presents a number of advantages for the Hospital; in particular, because it cuts down the costs of the pre-admission clinic.

We assume that this method is likely to increase the parents’ satisfaction because the pre-anesthesia visit is performed by the same anesthetist who will administer the anesthesia, and because it is likely to cut down the indirect costs for the family.

Questions:

1. What is the “savings economic value” (savings in terms of days of work, transport costs, family stress)? Check list:
   1. less than € 20
   2. from € 20 to € 50
   3. from € 50 to € 100
   4. from € 100 to € 200
   5. from € 200 to € 250
2. How much are you willing to pay to avoid an unnecessary access to the pre-admission clinic?
   1. less than € 20
   2. from € 20 to € 50
   3. from € 50 to € 100
   4. from € 100 to € 200
   5. from € 200 to € 250
3. Had you been required to go to the pre-admission clinic, would you have lost a day of work?
   1. yes
   2. no
4. What is your personal opinion on the one-stop anesthesia?
   1. poor
   2. fair
   3. good
   4. excellent
